# Phase I clinical evaluation of ^99m^Tc-labeled Affibody molecule for imaging HER2 expression in breast cancer

**DOI:** 10.7150/thno.86770

**Published:** 2023-09-04

**Authors:** Olga Bragina, Vladimir Chernov, Mariia Larkina, Anstasiya Rybina, Roman Zelchan, Eugeniy Garbukov, Maryam Oroujeni, Annika Loftenius, Anna Orlova, Jens Sörensen, Fredrik Y. Frejd, Vladimir Tolmachev

**Affiliations:** 1 Department of Nuclear Therapy and Diagnostic, Cancer Research Institute, Tomsk National Research Medical Center, Russian Academy of Sciences, Tomsk, Russia.; 2 Research Centrum for Oncotheranostics, Research School of Chemistry and Applied Biomedical Sciences, Tomsk Polytechnic University, Tomsk, Russia.; 3 Department of Pharmaceutical Analysis, Siberian State Medical University, 634050 Tomsk, Russia.; 4 Department of Immunology, Genetics and Pathology, Uppsala University, 752 37 Uppsala, Sweden.; 5 Affibody AB, Solna, Sweden.; 6 Department of Medicinal Chemistry, Uppsala University, Uppsala, Sweden.; 7 Radiology and Nuclear Medicine, Department of Surgical Sciences, Uppsala University, Uppsala, Sweden.

**Keywords:** HER2, Affibody, ^99m^Tc, ZHER2:41071, SPECT, Phase I

## Abstract

The determination of tumor human epidermal growth factor receptor type 2 (HER2) status is of increasing importance with the recent approval of more efficacious HER2-targeted treatments. There is a lack of suitable methods for clinical *in vivo* HER2 expression assessment. Affibody molecules are small affinity proteins ideal for imaging detection of receptors, which are engineered using a small (molecular weight 6.5 kDa) nonimmunoglobulin scaffold. Labeling of Affibody molecules with positron emitters enabled the development of sensitive and specific agents for molecular imaging. The development of probes for SPECT would permit the use of Affibody-based imaging in regions where PET is not available. In this first-in-human study, we evaluated the safety, biodistribution, and dosimetry of the ^99m^Tc-ZHER2:41071 Affibody molecule developed for SPECT/CT imaging of HER2 expression.

**Methods**: Thirty-one patients with primary breast cancer were enrolled and divided into three cohorts (injected with 500, 1000, or 1500 µg ZHER2:41071) comprising at least five patients with high (positive) HER2 tumor expression (IHC score 3+ or 2+ and ISH positive) and five patients with low (IHC score 2+ or 1+ and ISH negative) or absent HER2 tumor expression. Patients were injected with 451 ± 71 MBq ^99m^Tc-ZHER2:4107. Planar scintigraphy was performed after 2, 4, 6 and 24 h, and SPECT/CT imaging followed planar imaging 2, 4 and 6 h after injection.

**Results**: Injections of ^99m^Tc-ZHER2:41071 were well tolerated and not associated with adverse events. Normal organs with the highest accumulation were the kidney and liver. The effective dose was 0.019 ± 0.004 mSv/MBq. Injection of 1000 µg provided the best standard discrimination between HER2-positive and HER2-low or HER2-negative tumors 2 h after injection (SUV_max_ 16.9 ± 7.6 *vs.* 3.6 ± 1.4, p < 0.005). The ^99m^Tc-ZHER2:41071 uptake in HER2-positive lymph node metastases (SUV_max_ 6.9 ± 2.4, n = 5) was significantly (p < 0.05) higher than that in HER2-low/negative lymph nodes (SUV_max_ 3.5 ± 1.2, n = 4). ^99m^Tc-ZHER2:41071 visualized hepatic metastases in a patient with liver involvement.

**Conclusions**: Injections of ^99m^Tc-ZHER2:41071 appear safe and exhibit favorable dosimetry. The protein dose of 1000 µg provides the best discrimination between HER2-positive and HER2-low/negative expression of HER2 according to the definition used for current HER2-targeting drugs.

## Introduction

Molecular recognition of human epidermal growth factor receptor type 2 (HER2) is exploited by a number of therapeutics for the treatment of breast cancer 1,2. An initial breakthrough was achieved using the unarmed monoclonal antibodies trastuzumab and pertuzumab for the treatment of patients with HER2-positive cancers as defined by immunohistochemistry (IHC) scores of 3+ or 2+ and *in situ* hybridization (ISH) showing gene amplification in biopsy samples 3, 4. This success was followed by the development of HER2-targeting antibody‒drug conjugates, which improved the survival of patients with progressive disease after two or more HER2-directed therapies 5, 6. The most impressive recent development is the approval of trastuzumab deruxtecan, which resulted in a significant increase in the progression-free and overall survival of patients, even those with HER2-low metastatic breast cancer (IHC score 1+ or 2+ and negative in ISH), who had received one or two previous lines of chemotherapy 7. Nevertheless, serious adverse reactions occurred in 20% of patients receiving trastuzumab deruxtecan, and it is approved only after failure of first-line therapies 8. To use these therapies, determination of the HER2 expression level in tumors is required 9. The level of HER2 expression is determined in biopsy samples, and only tumors with sufficient expression of this molecular target (immunohistochemistry (IHC) score 3+ or 2+ and *in situ* hybridization (ISH) showing gene amplification) are considered HER2-positive, *i.e.,* suitable for first-line HER2-targeted therapies 10. Recent data from studies with trastuzumab deruxtecan now show that even tumors with lower HER2 expression levels respond to treatment, and a new definition of HER2-high (IHC score 3+ or 2+ and ISH-positive), HER2-low (IHC score 1+ or 2+ and negative in ISH) and HER2-negative (IHC score 0) is emerging 7. The expression level of HER2 may change during metastasis and disease progression 11, 12, and new invasive biopsies would thus be required to assess relapsing tumors. Radionuclide molecular imaging of HER2 expression could address issues of spatial and temporal heterogeneity of HER2 expression, enabling better selection of patients for targeted therapy and avoiding the need for invasive biopsies 13. A promising format of tracers for molecular visualization of HER2 is engineered scaffold proteins (ESP) 13 since their small size allows efficient visualization of HER2 on the day of injection. Affibody molecules are small ESP proteins (molecular weight 6.5 kDa) and have been engineered to bind to HER2 with an affinity of 29 pM 14. Phase I/II studies have demonstrated that PET/CT imaging using the ^68^Ga-labeled Affibody molecule ABY-025 permits discrimination between breast tumors with high and low HER2 levels 15 3-4 h after injection and predicts the outcome of trastuzumab therapy 16. A number of other PET tracers based on derivatives of HER2-targeting Affibody molecule showed the capacity to discriminate breast and gastric cancers with high and low HER2 expression in clinical trials 17-20. It should be noted that PET imaging facilities are primarily available in high-income countries, while the availability in low-income and middle-income countries is limited due to economic and logistical issues 21, whereas SPECT/CT cameras are more accessible 21. The use of SPECT/CT for radionuclide imaging of HER2 expression in geographic regions where PET/CT is not available might expand the field of imaging-guided treatment of breast cancer, making it cheaper and more accessible. Importantly, modern SPECT/CT scanners permit corrections for scatter, attenuation and partial volume, enabling *in vivo* quantification accuracy of 5% 22, which makes them suitable for molecular imaging. In principle, ABY-025, which contains a DOTA chelator, is suitable for labeling with ^111^In 23. However, the use of a ^99m^Tc-labeled tracer would be ideal regarding resolution and sensitivity and from logistics and economic points of view. For labeling with ^99m^Tc, a new variant of an Affibody molecule, ZHER2:41071, with modifications in the scaffold, including incorporation of the chelating sequence GGGC at the C-terminus, was developed 24. This sequence can be directly encoded into the primary structure of the Affibody molecule, which excludes the conjugation of a chelator as an additional production step 24 and makes the production of the tracer less costly. The labeling of Affibody molecules containing this chelator is straightforward and stable 24, 25. In addition, the use of GGGC as a chelator provided lower retention of ^99m^Tc in kidneys compared with the use of homologous peptide-based chelators 25. The modification of the scaffold and the replacement of the nuclide and a chelator may, however, substantially alter the biodistribution profile of Affibody molecules 26 and change absorbed doses. In addition, previous studies have demonstrated that an injection of 427 ± 19 µg ^68^Ga-ABY-025 enables higher contrast of imaging of hepatic metastases compared with an injection of 78 ± 8 µg 15. To date, it has remained unclear whether an injected mass of approximately 400 µg is optimal for HER2-imaging Affibody molecules or whether imaging could be further improved by increasing the injected mass of ESP.

In this first-in-human study (ClinicalTrials.gov Identifier: NCT05203497), ^99m^Tc-ZHER2:41071 was evaluated in patients with primary HER2-positive and HER2-low or HER2-negative breast cancer as discriminated based on standard IHC/ISH biopsy results.

The primary objectives of the study were to obtain information concerning the safety and tolerability of ^99m^Tc-ZHER2:41071 after a single intravenous injection, to assess the distribution of ^99m^Tc-ZHER2:41071 in normal tissues and in tumors over time and to evaluate the dosimetry of ^99m^Tc-ZHER2:41071.

The secondary objective was to compare the tumor imaging data with HER2 expression data obtained by immunohistochemistry (IHC) and fluorescence *in situ* hybridization (FISH) analysis of biopsy samples.

Taking into account that the clinical data concerning imaging of HER2 using the Affibody molecule ^68^Ga-ABY-025 have demonstrated that uptake both in tumors and in normal tissues, as well as dosimetry, depends on the injected protein mass 15,27 and that only doses up to 500 µg were tested, the distribution after injection of 500, 1000, and 1500 µg ^99m^Tc-ZHER2:41071 was studied.

## Methods

### Patients

This was a prospective, open-label, nonrandomized phase I diagnostic study in patients with untreated primary breast cancer (ClinicalTrials.gov Identifier: NCT05203497). The study protocol was approved by the Scientific Council of Cancer Research Institute and Board of Medical Ethics, Tomsk National Research Medical Center of the Russian Academy of Sciences (protocol №13, 09.11.2021), and all patients signed a written informed consent form.

Inclusion criteria were: age of over 18 years; diagnosis of primary breast cancer stage I-II; HER2 status previously determined on material from the primary tumor; volumetrically quantifiable tumor lesions on US, CT or MRI, with at least one lesion > 2.0 cm in greatest diameter; hematological, liver and renal function test results within normal limits; a negative pregnancy test for all patients of childbearing potential; patient capable of undergoing the diagnostic investigations to be performed in the study; and informed consent.

Exclusion criteria were concomitant nonbreast malignancy; active current autoimmune disease or history of autoimmune disease; active infection or history of severe infection within the previous 3 months (if clinically relevant at screening); known HIV-positive or chronically active hepatitis B or C; administration of another investigational medicinal product within 30 days of screening; ongoing toxicity > grade 2 from previous standard or investigational therapies, according to US National Cancer Institute's guidelines.

The level of expression of HER2 in biopsy samples was determined by immunohistochemistry using Herceptest (DAKO). In the case of a score of 2+, fluorescent *in situ* hybridization (FISH) with the LSI HER2/neu (17q12)/CEP17 probe (Leica) was used to assess HER2 amplification. The tumors were classified as HER2-positive in cases of IHC score 3+ or IHC score 2+ and FISH-positive and HER2-low or negative if below these scores. Lymph node (LN) metastases were confirmed by histology using core biopsy or cytology using fine-needle biopsy in all patients.

**Figure 1:**
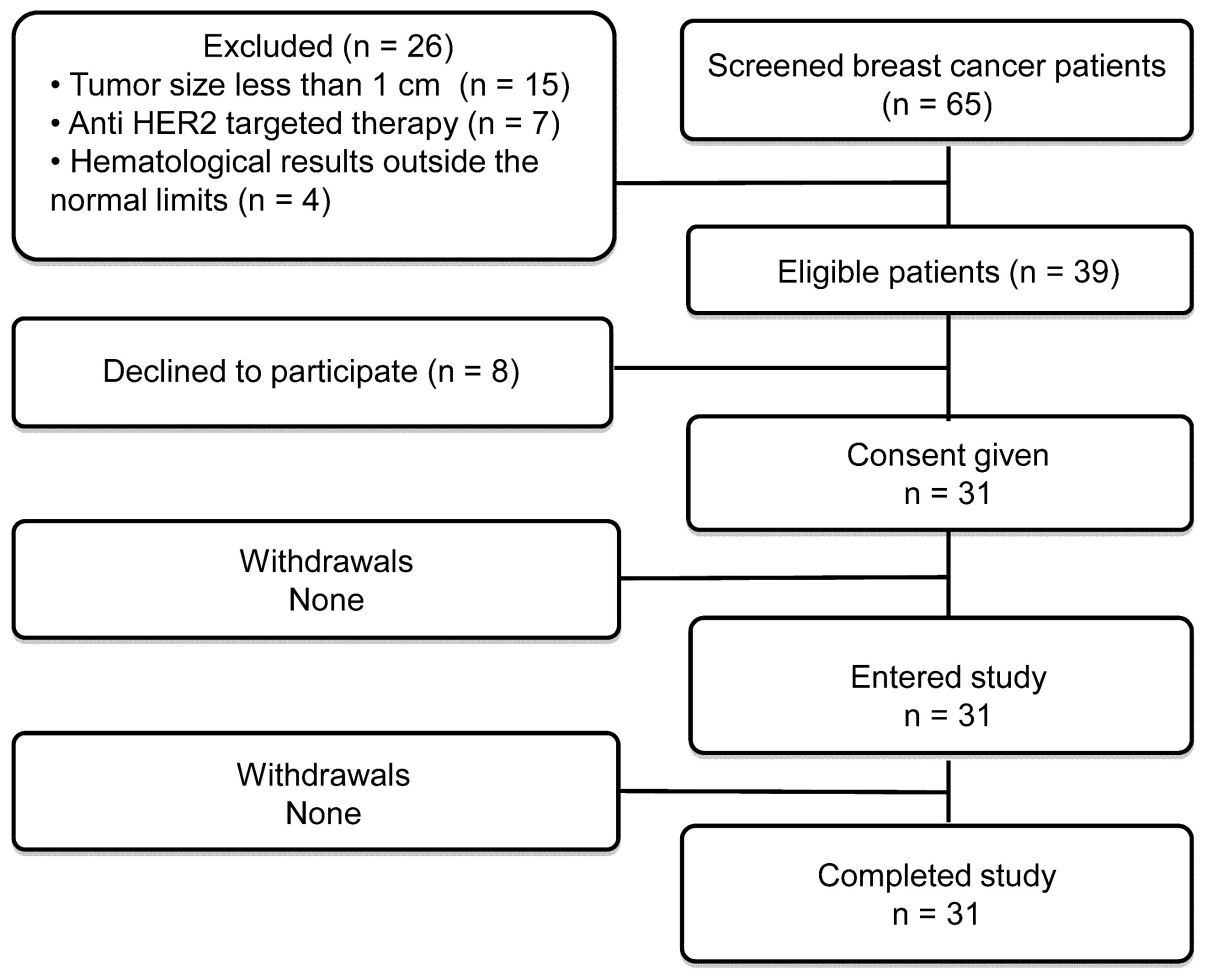
Flow diagram according to Standards for Reporting of Diagnostic Accuracy Studies (STARD).

Thirty-one patients were enrolled (Table 1; Figure 1). Patients were divided into three cohorts injected with 500 μg (patients 1-10), 1000 μg (patients 11-21) or 1500 μg (patients 22-31) of ^99m^Tc-ZHER2:41071. The third cohort (1500 μg) was added through an amendment after results became available from the first two cohorts with lower doses. Each group of patients included at least five patients with HER2-positive and five patients with HER2-low or HER2-negative tumors. There were six HER2-positive breast cancer patients in the 1000 μg cohort. One patient in this cohort had two synchronous HER2-low tumors (IHC score 1+), one in the left breast and one in the right breast. Recruitment of patients into a cohort with a higher injected dose was initiated no earlier than accomplishment of the safety evaluation of the preceding cohort with a lower injected dose. In each cohort, patients were enrolled consecutively until the required numbers for HER2-high and HER2-low or negative tumors were achieved.

For all patients, biopsy sampling, mammography (Giotto Image), bone scan (Siemens E.Cam 180) using ^99m^Tc-pyrophosphate, chest CT (Siemens Somatom Emotions 16 ECO) and ultrasound imaging of the breast, regional lymph nodes and liver (GE LOGIQ E9) were performed. The size of the primary tumor and metastatic lymph nodes were measured using ultrasound. For patient 15, a contrast-enhanced abdominal CT was performed additionally. Physical examination, clinical lab analysis and ECG were additionally performed immediately before imaging and after the last scan.

### Imaging Protocol

Labeling of ^99m^Tc-ZHER2:41071 was performed under aseptic conditions according to the method described earlier 24. For this study, a labeling kit was manufactured by the contract manufacturing organization Almac Sciences (Craigavon, Northern Ireland, United Kingdom). The kit included a vial with a freeze-dried labeling mixture and vials with freeze-dried ZHER2:41071 (1000 µg). Each vial of labeling mixture contained 82.5 µg of tin(II) chloride dihydrate (Merck, Darmstadt, Germany), 5.5 mg of gluconic acid sodium salt (Sigma‒Aldrich, Saint Louis, MO) and 102.8 µg of ethylenediaminetetraacetic acid-di-sodium salt dihydrate (EDTANa_2_ × 2H_2_O) (Merck, Darmstadt, Germany). The contents of the vials were shown to be nontoxic, sterile and apyrogenic.

For labeling, 150 µL sterile phosphate-buffered saline (PBS) was added to a vial with ZHER2:41071. The vial content was mixed and checked visually to ensure that the content was completely dissolved, and no particles or opalescence was observed.

The ^99m^Tc-generator eluate (400 µL) was added to the reaction mixture, and the vial content was vortexed. The content of the vial was extracted by a sterile syringe through the rubber stopper and injected into a vial containing ZHER2:41071. The vial was vortexed and incubated for 60 min at 70 °C and then cooled for 2 min. A sterile isotonic solution (0.5 mL) was added, the contents were mixed, and the mixture was passed through a sterile filter (Sartorius, 0.22 µm) into a vial for the sterile product. A droplet of the reaction mixture was applied on an ITLC strip, and the strip was developed in PBS. The distribution of activity along the strip was measured using an Elysia-raytest miniGita ITLC scanner (Elysia-raytest GmbH, Straubenhardt, Germany). Based on the labeling yield and activity in the sterile vial, an exact amount of transferred ZHER2:41071 was calculated. The content of another ZHER2:41071 vial was dissolved in a sterile isotonic solution, and the correct volume was added to the sterile vial to obtain the required injected mass of ZHER2:41071 (500, 1000 or 1500 µg). The content of the sterile vial was diluted with 5 mL of sterile isotonic solution, and the syringe for injection was filled. The activity in the syringe was measured before and after injection.

The yield was 97 ± 1%. The injected activity was 451 ± 71 MBq. Monitoring of safety, thorough assessment of vital signs, ECG and physical examination were performed during imaging visits (0-24 h after injection) and 3-7 days after injection.

Imaging was performed using a Siemens Symbia Intevo Bold scanner equipped with a high-resolution low-energy collimator. Anterior and posterior planar whole-body imaging (at a scan speed of 12 cm/min, 1024 × 256 pixel matrix) was performed at 2, 4, 6 and 24 h after injection of ^99m^Tc-ZHER2:41071. SPECT/CT scans (SPECT: 60 projections, 20 seconds each, stored in 256 × 256 pixel matrix/CT: 130 kV, effective 36 mAs) covering the area from neck to liver were performed 2, 4 and 6 hours after injection of ^99m^Tc-ZHER2:41071. SPECT images were reconstructed using the xSPECT (Siemens) protocol based on the ordered subset conjugate gradient (OSCG) method (24 iterations, 2 subsets) with a 3D Gaussian FWHM 10 mm filter (Soft Tissue). The images were processed using the proprietary software package Syngo.via (Siemens).

Blood samples were obtained 5, 10, 15, 20, 30 and 50 min after injection if the patient's condition permitted. Small (500 µL) aliquots were taken, and activity was measured using an automated gamma-spectrometer with a NaI (TI) detector (Wizard 1480, Perkin Elmer, Waltham) to determine the activity in blood during the distribution phase.

Dosimetry was evaluated as described in 28. Briefly, regions of interest (ROIs) were drawn over organs of interest and the whole body on the anterior and posterior whole-body images of patients injected with ^99m^Tc-ZHER2:41071; a geometric mean of counts at 2, 4, 6 and 24 h was calculated for each ROI. To assess kinetics in blood, an ROI was placed over the heart. For quantification, the known activity of ^99m^Tc in a water-filled phantom in combination with Chang's correction was used. Data were fitted by single exponential functions, and residence time was calculated as areas under fitted curves using Prism 8 (GraphPad Software, LLC). Absorbed doses were calculated by OLINDA/EXM 1.1 using Adult Female phantom.

Maximal standard uptake values (SUV_max_) were calculated in primary tumors, lymph nodes, liver, and liver metastases, as well as for contralateral symmetric regions of the breast and contralateral symmetric regions for lymph node metastases for determination of tumor-to-contralateral breast and lymph node metastases to background ratios at 2, 4 and 6 h after injection. For patient #15, SUV_max_ was also calculated in metastasis-free areas in the liver for determination of the liver metastases-to-liver ratio.

### Statistics

Values are reported as the mean ± SD. Differences between uptakes in organs at different injected masses of the protein were analyzed using one-way analysis of variance (ANOVA). The nonparametric Mann-Whitney U test was used to determine whether the differences between uptake for HER2-positive and HER2-low or HER2-negative tumors were significant. A 2-sided P value of less than 0.05 was considered significant. Paired t tests were used to analyze differences between uptake in organs at different time points. A linear regression analysis was performed to determine if there was a correlation (the slope was significantly nonzero) between the HER2 expression level determined by IHC/FISH and tumor uptake. The sensitivity and specificity of discrimination between HER2-positive tumors and tumors with low expression or HER2-negative tumors using imaging after injection of 1000 µg ^99m^Tc-Z41071 was evaluated by ROC analysis. The statistical analysis was performed using Prism 9 for Windows (Graph Pad Software, LLC, Boston, MA).

## Results

All injections were well tolerated. Two patients (in cohorts injected with 500 and 1500 µg) had a transient blood pressure drop, which was deemed not related to ^99m^Tc-Z41071 injection by the investigator. No clinically significant changes were registered in terms of vital signs or results of blood and urine analyses before and after ^99m^Tc-Z41071 injection.

^99m^Tc-ZHER2:41071 was cleared rapidly from the blood. The half-life of the first (distribution) phase was 4.7 ± 1.4 min, and the half-life of the second (elimination) phase was 3.7 ± 1.5 h.

**Figure 2:**
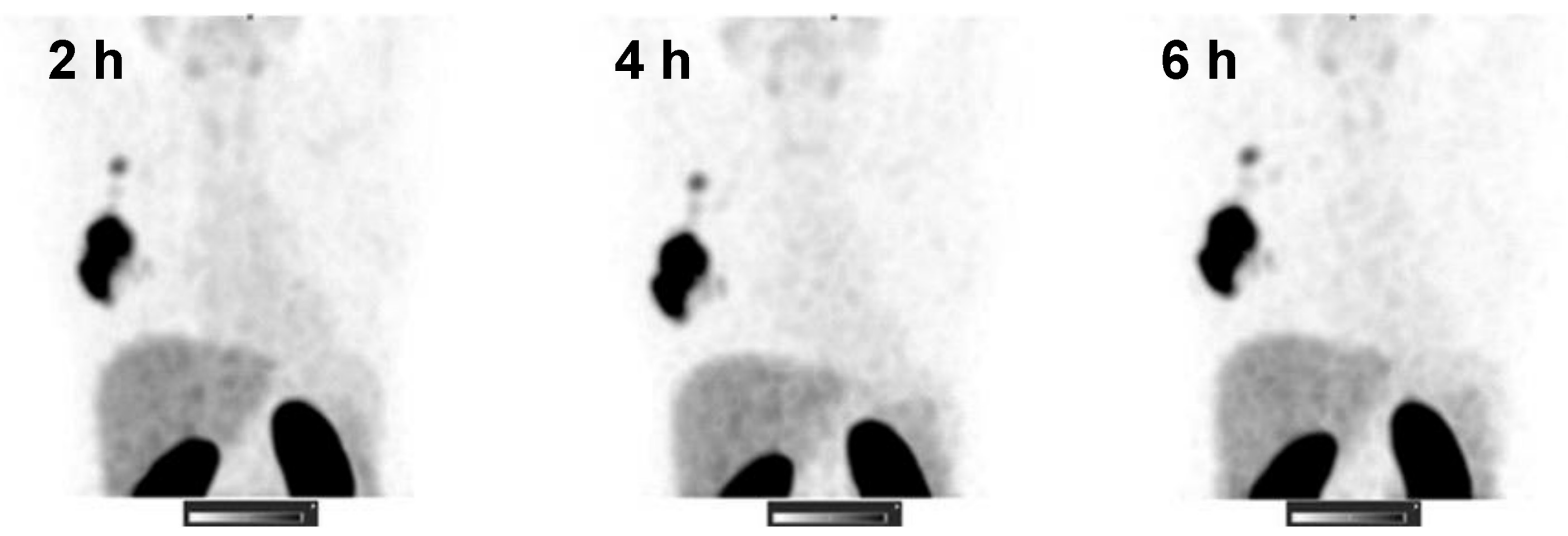
SPECT images (MIP) at 2, 4, and 6 h after injection of 1000 µg ^99m^Tc-ZHER2:41071 (Patient 16). Linear SUV scale from 0 to 15.

**Figure 3:**
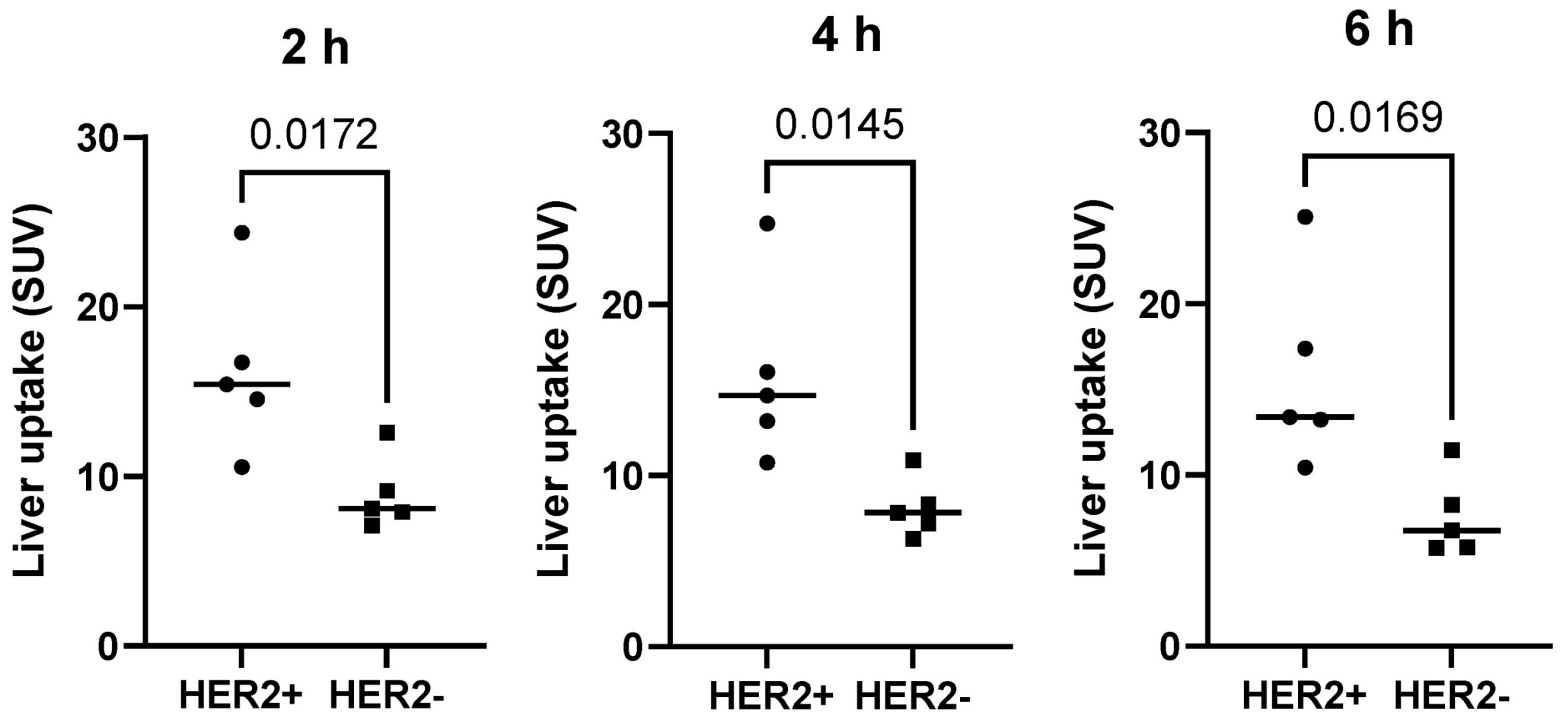
Uptake (SUV) in the liver in patients with different HER2 expression levels after injection of 500 µg ^99m^Tc-ZHER2:41071. The statistical significance was evaluated using the Mann-Whitney U test.

The kidneys and liver were the normal organs with the highest uptake activity (Figure 2, Table 2). Other organs with elevated uptake were the breast and lung. There were no significant differences in uptake in these organs (expressed as %ID/organ) between cohorts injected with different protein doses, except uptake in breast 24 h after injection, which was significantly lower in patients injected with 1500 µg compared with 500 µg. However, comparison of SUV (*i.e.,* taking into account the body mass) showed that the injection of 1500 µg resulted in significantly (P < 0.05, one-way ANOVA with Tukey correction for multiple comparisons) lower hepatic uptake compared with the uptake after injection of 500 µg. Interestingly, the hepatic uptake (SUV) of ^99m^Tc-ZHER2:41071 was significantly higher (P < 0.05, Mann-Whitney U test) in patients with HER2-positive primary tumors than in patients with HER2-low tumors at all time points for the cohort injected with 500 µg (Figure 3). In other cohorts, there was no significant difference between hepatic uptake in patients with HER2-positive and HER2-negative or HER2-low primary tumors.

The clearance of ^99m^Tc-ZHER2:41071 from normal tissues depended on the injected mass. For the cohort injected with 500 µg, the uptake was significantly (P < 0.05, paired t test) reduced between 2 and 4 h after injection in the brain, noninvolved breast, stomach, thymus and muscles. Uptake at 6 hours after injection was additionally reduced in the gall bladder and small intestines. For the cohort injected with 1000 µg, a significant reduction in uptake between 2 and 4 h was found in muscles, with an additional reduction in the noninvolved breast, stomach and lungs by 6 hours after injection. In the case of injection of 1500 µg, a significant reduction in uptake between 2 and 4 h was found in the brain, noninvolved breast, small intestines, stomach, liver, lung, pancreas, thymus and muscles. By 6 h, additional clearance from the gall bladder was found.

Estimated absorbed doses are presented in Table 3. The highest absorbed organ doses were found in kidneys and adrenals. Lower but noticeable absorbed doses were observed in the pancreas, ovary, gall bladder wall, liver, and spleen. The absorbed doses to thyroids, ovaries, thymus and stomach wall were higher (P < 0.05) for the 1500 µg cohort than for the 500 µg cohort, but the difference was modest. The total effective doses were 0.017 ± 0.003, 0.018 ± 0.005 and 0.021 ± 0.004 mSv/MBq for patients injected with 500 µg, 1000 µg and 1500 µg of ^99m^Tc-ZHER2:41071, respectively.

**Figure 4:**
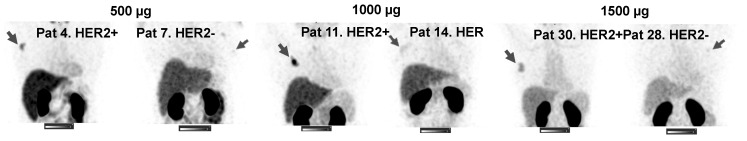
SPECT images of primary tumors (MIP) 2 h after injection of 500, 1000 or 1500 µg ^99m^Tc-ZHER2:41071. Linear SUV scale from 0 to 15. Arrows point at tumors.

**Figure 5:**
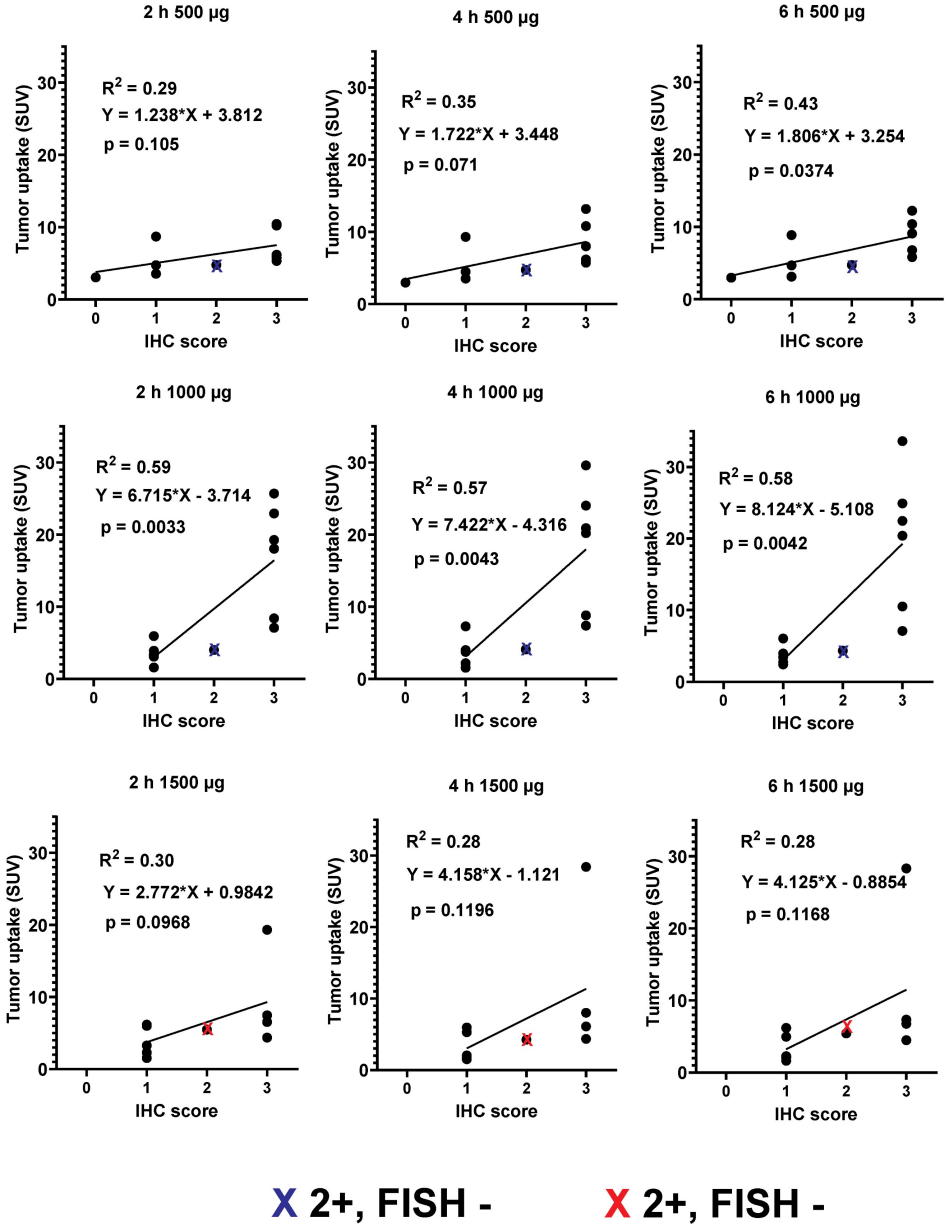
Relationship between data concerning the expression of HER2 in biopsy samples and SUV _max_ at different time points after injection of 500, 1000 and 1500 µg ^99m^Tc-ZHER2:41071. Red crosses mark data for FISH-positive tumors with IHC score 2+, blue crosses mark data for FISH-negative tumors with IHC score 2+.

Primary tumors with both high and low/negative HER2 expression levels were visualized in this study. Representative images of primary tumors are presented in Figure 4. The quantitative data concerning uptake in primary tumors with different HER2 expression levels are presented in Table 4 and Figure 5. From 2 h to 6 h, the tumor uptake was constant (no significant changes in the tumor uptake measured as SUV_max_ at different time points) for any injected protein mass. After injection of 500 µg ^99m^Tc-ZHER2:41071, a significant difference (P < 0.05, Mann-Whitney U test) between uptake in tumors with high and low or negative HER2 expression was observed only at 6 h after injection. After injection of 1500 µg, there was no significant difference at any time point. In contrast, injection of 1000 µg resulted in a highly significant difference (P < 0.005, Mann-Whitney U test) between uptake in tumors with high and low HER2 expression. Receiver operating characteristic (ROC) analysis demonstrated that imaging using 1000 µg afforded the best performance with an AUC of 1.0 at 2 h after injection (sensitivity of 100% and specificity of 100%; SUV cutoff > 6.5). In the case of an injected dose of 500 µg, the accuracy was 80% (AUC of 0.88%; sensitivity of 100% and specificity of 80%; cut off > 5.061). In the case of an injected dose of 1500 µg, the accuracy was 60% (AUC of 0.84%; sensitivity of 100% and specificity of 60%; cutoff-off > 3.83). Furthermore, a regression analysis (Figure 5) demonstrated that there was a significant (P < 0.005) linear correlation between the level of HER2 expression determined using IHC and the uptake (SUV _max_) at all time points after injection of 1000 µg ^99m^Tc-ZHER2:41071. A significant correlation (P < 0.05) was also found for SUV _max_ values obtained 6 h after injection of 500 µg ^99m^Tc-ZHER2:41071.

**Figure 6:**
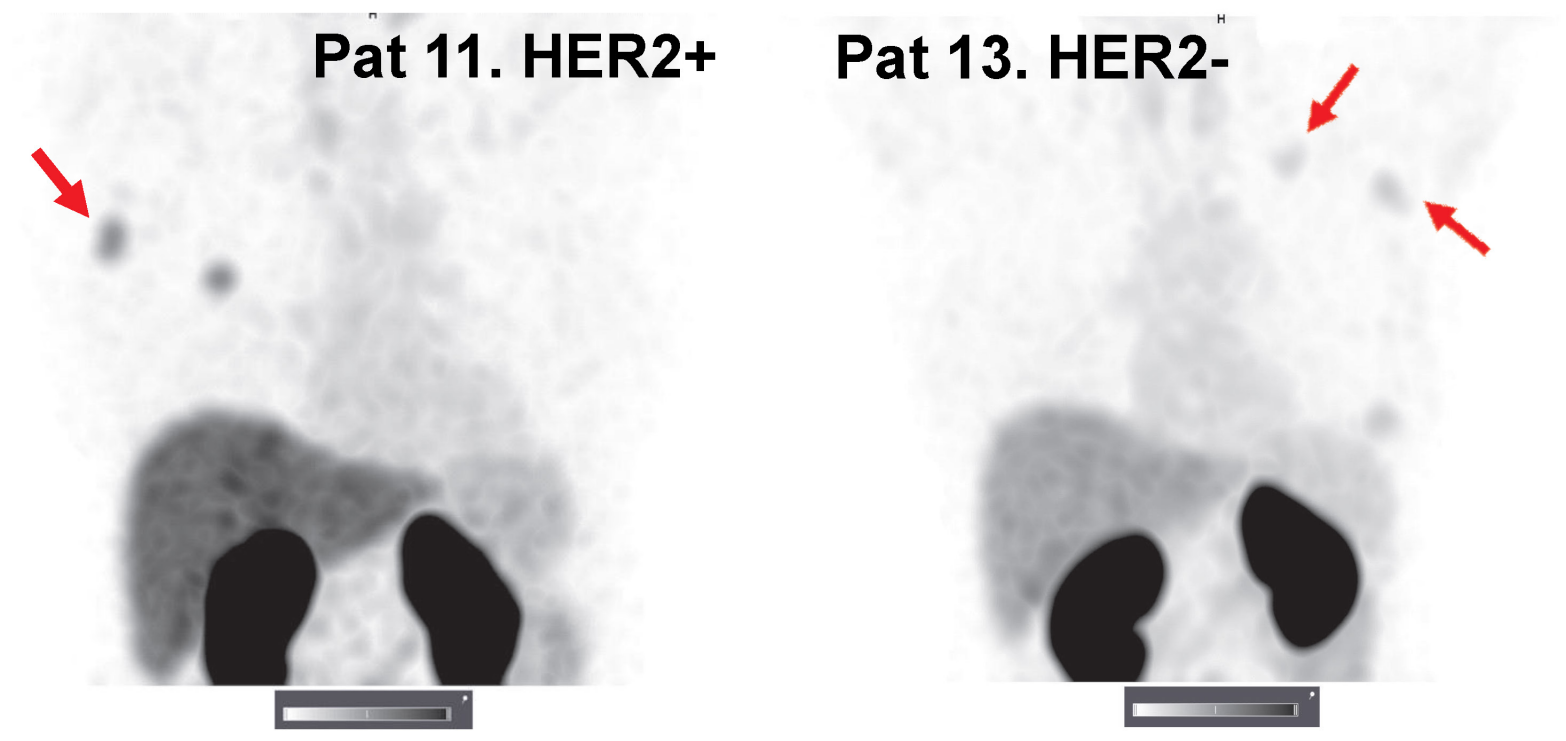
Representative SPECT/CT images of lymph node metastases 2 h after injection of 1000 µg ^99m^Tc-ZHER2:41071. Linear SUV scale from 0 to 15. Arrows point at lymph node metastases.

Imaging using ^99m^Tc-ZHER2:41071 permitted visualization of all known lymph node metastases. The number of lymph node metastases was limited, and most were smaller than the primary tumors. Nevertheless, our statistical analysis showed that the uptake in HER2-positive metastases (6.9 ± 2.4, n = 4) was significantly (P < 0.05) higher than that in HER2-low metastases (3.5 ± 1.2, n = 4) 2 h after injection of 1000 µg (Figure 6).

Quantitative data concerning tumor-to-contralateral site ratios for primary tumors with different HER2 expression levels and for HER2-positive lymph node lesions, where the number of lesions is sufficient for statistical treatment, are shown in Tables 5 and 6. Injection of 1000 µg ^99m^Tc-ZHER2:41071 also tended to provide higher average values in HER2-positive tumors, but the difference was not statistically significant. In contrast, 1000 µg ^99m^Tc-ZHER2:41071 was the only injected mass providing a statistically significant (P < 0.005, Mann-Whitney U test) difference between the ratios for HER2-positive and HER2-low/negative tumors at all time points. Injection of 500 µg ^99m^Tc-ZHER2:41071 resulted in a statistically significant (P < 0.05, Mann-Whitney U test) difference between values for HER2-positive and HER2-low/negative lesions only 6 h after injection. In the case of HER2-positive lymph node metastases, injection of 500 µg ^99m^Tc-ZHER2:41071 resulted in a significantly (P <0.05, one-way ANOVA with Tukey correction) higher value compared to injection of 1000 µg. However, there was no significant difference at later time points.

One patient with a HER2-positive primary tumor had several hepatic metastases confirmed by contrast-enhanced CT. Injection of 1000 µg ^99m^Tc-ZHER2:41071 enabled clear visualization of all metastases (Figure 6). Remarkably, some hepatic metastases had much higher uptake of the tracer than the primary tumor. The average liver metastasis-to-liver ratio was 4.0 ± 2.4 at 2 h after injection.

## Discussion

Recent advances in HER2-targeted therapy have prompted the need for increased understanding of HER2 expression levels, ideally in each tumor lesion in each patient. It is important to consider the availability and ease of use of the method to become broadly useful. Visualization and quantification of the expression of molecular targets for precision treatment of cancer within theranostics is typically associated with the use of PET 29 because of well-established accurate measurements of radioactivity concentrations *in vivo*, which is provided by this technique. However, the evolution of SPECT/CT cameras makes this modality potentially useful for quantitative molecular imaging as well 22. The costs for SPECT/CT scanners and appropriate radiochemical laboratories are lower than those for corresponding PET/CT facilities. Thus, recent development of tracers for the single-photon imaging of PSMA and FAPI was motivated by the lower cost and wider availability of SPECT 30, 31, paving the way for the introduction of theranostics in geographies currently underserved with PET/CT facilities. Importantly, a supply of inexpensive ^99^Mo/^99m^Tc generators is established for any operating SPECT facility. In addition to the better availability of SPECT scanners and the nuclide, the use of ^99m^Tc offers the advantages of a potential kit formulation permitting reliable labeling. This study demonstrated reproducible and efficient labeling of the Affibody ZHER2:41071 using a two-vial kit. The high radiochemical purity of ^99m^Tc-ZHER2:41071 permitted its clinical use without any additional purification. Our experience shows that the development of a one-vial kit for labeling Affibody molecules using peptide-based cysteine-containing chelators is also relatively simple 32. This feature of the tracer supports its implementation in radiochemistry laboratories with basic equipment suitable for ^99m^Tc labeling in low- and middle-income countries.

This study demonstrated that injections of ^99m^Tc-ZHER2:41071 are tolerable, and no clinically significant adverse events were registered. Before the start of this clinical trial, an extended single-dose toxicity study was performed. Sprague‒Dawley rats were injected with a dose of 6 mg/kg, which exceeds the maximum mass per body weight injected in the clinical trial by approximately 240-fold. No difference in general conditions and weight, hematology parameters, coagulation system, clinical chemistry parameters, or pathomorphology was found between the treated animals and control groups. The safety and tolerability of ^99m^Tc-ZHER2:41071 was confirmed in the present clinical trial. Furthermore, Liu and coworkers injected five patients with two 1 mg doses of ^18^F-labeled anti-HER2 Affibody molecules within one week. No toxicity or side effects were reported in that study 20. The effective dose for ^99m^Tc-ZHER2:41071 (between 0.017 ± 0.003 and 0.021 ± 0.004 mSv/MBq) was similar to the doses associated with the injection of ^68^Ga- or ^18^F-labeled HER2-targeting Affibody molecules (0.023-0.028 mSv/MBq) 18, 20, 27. The dose burden for ^99m^Tc-ZHER2:41071 was somewhat lower than that for single-domain antibodies (0.406 mGy/MBq for ^68^Ga-labeled 33 and 0.06-0.129 mSv/MBq for ^99m^Tc-labeled 34, 35 variants) and much lower than that for full-size IgG labeled with ^98^Zr (0.38 to 0.61 mSv/MBq) 36, 37, 38. It should be taken into account that the injected activity in this study was selected to provide a sufficiently high signal for distribution measurements even 24 h after injection for dosimetry. For routine diagnostics, the injected activity might be reduced by at least 200 MBq. In that case, even multiple injections could be performed with an acceptable dose burden, *i.e.,* 3.7 mGy per investigation.

An unexpected finding in this study was a higher hepatic uptake in patients with HER2-positive primary tumors compared with patients with HER2-low or HER2-negative ones when 500 µg was injected (Figure 3). There might be several mechanisms resulting in an elevated uptake of HER2-binding Affibody molecules in the liver, some depending on direct interactions with HER2 expressed on hepatocytes and some independent of this interaction. It is known that hepatocytes express some HER2 39, but the specific binding would not explain the association of hepatic uptake and HER2 expression in the primary lesion. A possible explanation for this phenomenon might be an elevated level of shed extracellular domain HER2 (sHER2) in the case of HER2 expression in the primary tumor 40. ^99m^Tc-ZHER2:41071 could bind to sHER2 in blood, and the complex might be transported into the liver. Increasing the injected protein mass to 1000 µg (higher level of nonlabeled protein) would lead to saturation of sHER2, causing reduced hepatic uptake of ^99m^Tc-ZHER2:41071 even in the case of high HER2 expression in the tumors. Interestingly, the impact of sHER2 on the biodistribution of ^68^Ga-ABY-025 was previously observed only in one patient of 23, who had an extremely high level of sHER2 41. That patient also had very high hepatic uptake. It cannot be excluded that different breast cancer populations have differences in sHER2 levels, and in this study, patients with primary tumors were included. Nonetheless, elevated hepatic uptake is undesirable for imaging hepatic metastases, especially small metastases, which would suggest the selection of a higher peptide dose for improved image contrast.

Studies with the ^68^Ga-labeled anti-HER2 Affibody molecule ABY-025 PET have shown that increases in injected mass from 78 ± 8 to 427 ± 19 µg improved the discrimination between liver background and tumor lesions, presumably due to decreasing the binding to hepatocytes and increasing the bioavailability of the tracer 15. This study demonstrated that an increase in the injected ^99m^Tc-ZHER2:41071 mass to 1000 µg improved the discrimination between HER2-positive and HER2-low primary tumors compared with an injected dose of 500 µg (Table 4). Nevertheless, it was unclear whether an increase in the injected mass beyond 1000 µg could improve imaging further, and this study was extended by including a cohort injected with 1500 µg. However, a further increase in the injected mass to 1500 µg resulted in a reduction in uptake in some tumors with high expression, potentially because of saturation of available receptors by unlabeled protein. The current study found that a maximum injected mass should be approximately 1000 µg. Although the sample size in this phase I study is limited, setting the SUVmax threshold level at 6.5 would enable baseline discrimination between HER2-high and HER2-low tumors in the case of injection of 1000 µg ^99m^Tc-ZHER2:41071. After injection of 1000 µg, the uptake (SUV _max_) of^ 99m^Tc-ZHER2:41071 in HER2-positive primary tumors (16.9 ± 7.6) 2 h after injection was on the same level or higher than the average uptake in HER2-positive primary breast cancer reported in clinical studies concerning other HER2-targeting Affibody molecules: ^68^Ga-NOTA-MAL-Cys-MZHER2:342 (2.16 ± 0.2) 17, ^18^F-AlF-NOTA-HER2-BCH (6.7 ± 4.3) and ^18^F-AlF-RESCA-HER2-BCH (7.8 ± 5.5) 20. This is also valid for other scaffold protein-based tracers for imaging HER2, such as ^99m^Tc-labeled ADAPT6 (4.7 ± 2.1) and DARPin G3 (3.5 ± 1.7) 42, as well as tracers based on single domain antibodies, such as ^68^Ga-NOTA-HER2-Nanobody 2Rs15 d (4.7 ± 4.8) 33, ^18^F-AlF-RESCA-MIRC213 nanobody (3.62 ± 1.56) 43 or ^99m^Tc-NM-02 nanobody (8.8 ± 2.6) 44. Obviously, it has to be taken into account that the reported values are obtained in Phase I studies with a limited number of patients, and the injected protein mass might not be optimized. Nevertheless, the numeric data indicate that the tumor-targeting properties of ^99m^Tc-ZHER2:41071 are at least as good as the properties of alternative imaging probes based on small proteins or, probably, better. Importantly, all scaffold-protein- and single domain antibody-based tracers, including ^99m^Tc-ZHER2:41071, have clear clinical advantages compared to HER2-imaging tracers based on intact IgG, which enable imaging only 4-5 days after injection 45 due to slow clearance from blood and have an appreciable rate of false-positive findings 46, most likely due to the enhanced permeability and retention (EPR) effect.

The injection of an increased mass of Affibody molecules may raise a reasonable question of whether this could affect the uptake of HER2-targeting drugs. However, anti-HER2 Affibody molecules bind to a HER2 epitope that is different from the epitopes of the most commonly used anti-HER2 monoclonal antibodies, trastuzumab and pertuzumab 47. Preclinical studies have shown that anti-HER2 Affibody molecules do not cross-block those monoclonal antibodies binding to HER2-expressing cells *in vitro* or *in vivo* 48, 49. It was also shown in a clinical trial that there was no significant effect of ^68^Ga-ABY-025 uptake in metastases of HER2-positive breast cancer when the patients were on trastuzumab therapy 15. Thus, it is unlikely that imaging using ^99m^Tc-ZHER2:41071 would be unfavorable for the use of HER2-targeting pharmaceutics.

**Figure 7:**
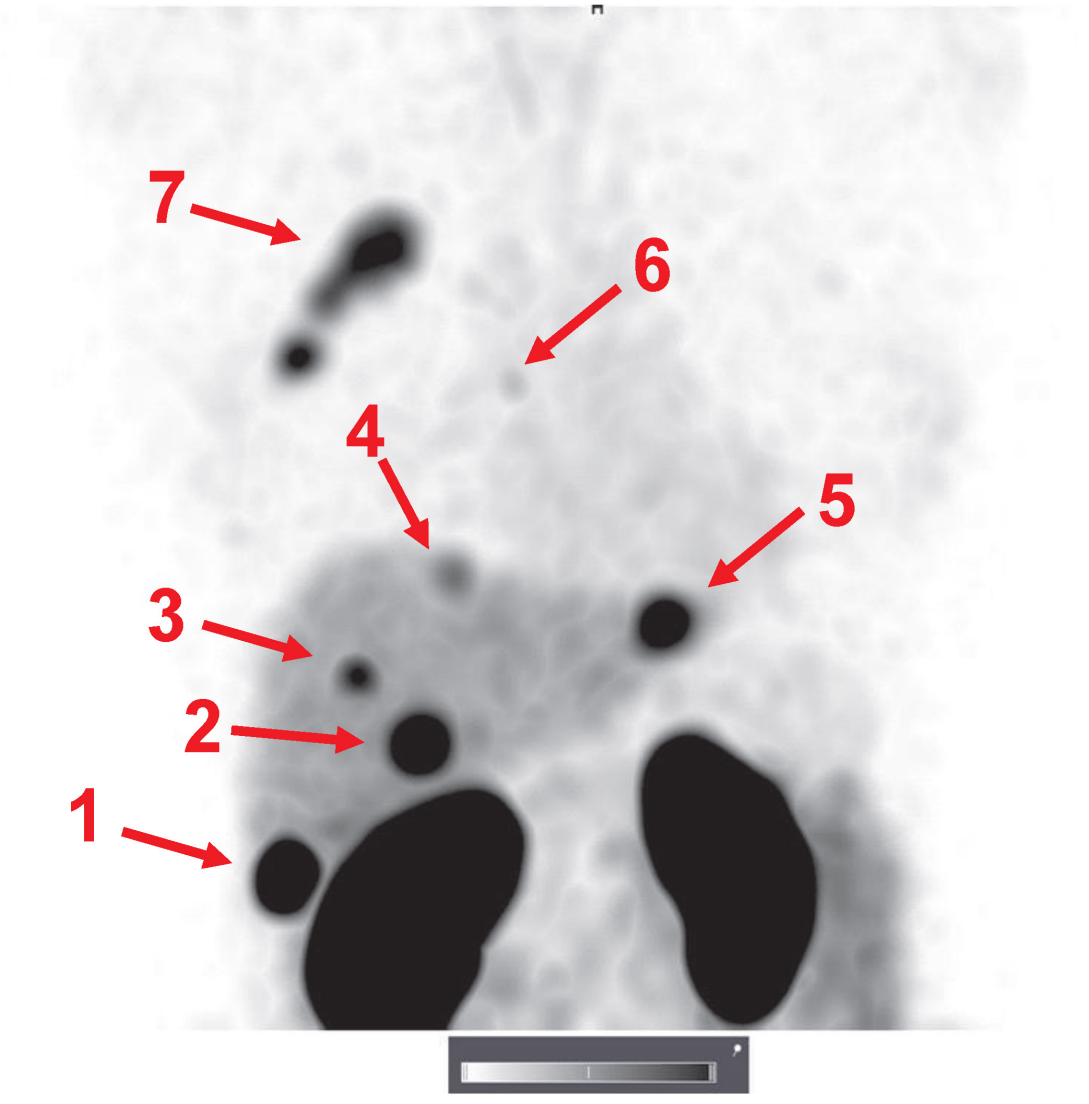
Visualization of hepatic metastases 2 h after injection of 1000 µg ^99m^Tc-ZHER2:41071. The uptake values (SUVs) for the hepatic metastases (arrows) were 1-75.7; 2-44.4; 3-22.1; 4-14.6; and 5-35.2. A metastasis in a parasternal lymph node (6) was also visualized (SUV max = 6.9). The uptake in the primary multicentric tumor (7) was 22.3. Linear SUV scale from 0 to 20.

An important observation was that the hepatic metastases were clearly visualized in patient 15 (Figure 7). Hepatic metastases occur frequently in HER2-positive breast cancer 50, and their visualization is a precondition for successful clinical translation for a HER2-specific imaging agent. Interestingly, the uptake level in several liver metastases far exceeded the uptake in the primary tumor. Most likely, this can be explained by the high blood perfusion of the liver.

**Figure 8:**
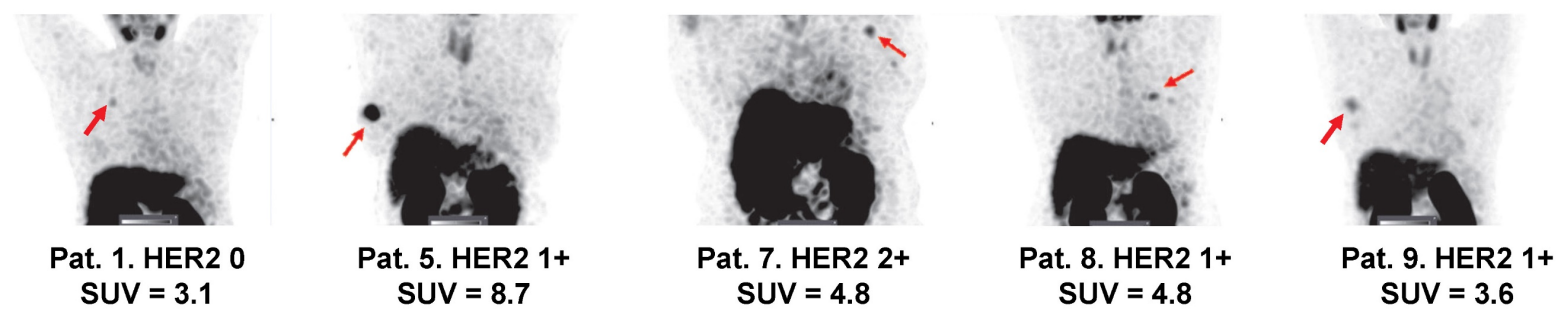
Visualization of primary tumors (MIP) with low HER2 expression 2 h after injection of 500 µg ^99m^Tc-ZHER2:41071. Linear SUV scale from 0 to 5. Arrows point at tumors. IHC score and tumor uptake (SUV_max_) are provided for each patient.

This study was designed to explore the impact of protein mass dose for detecting HER2 expression in light of current recommendations for the selection of first-line HER2-targeting therapies 10. Such therapies are recommended for cases when HER2 expression is 3+ or 2+ and ISH positive. Recent studies 7, 51 have shown that even patients with tumors with lower HER2 expression might benefit from treatment with the trastuzumab-deruxtecan conjugate. The trastuzumab-deruxtecan conjugate is, however, approved for the treatment of patients after the failure of two or more other treatments 8. The results of this study show that patients might be selected for first-line treatments using ^99m^Tc-ZHER2:41071-mediated SPECT/CT imaging. Apparently, it would be attractive to use the same tracer to select patients for trastuzumab-deruxtecan conjugate treatment, *i.e.,* to discriminate between tumors with an IHC score of 0 from tumors with a score of 1+ or higher. Importantly, tumors with low IHC scores still express HER2. Cross-calibration experiments have demonstrated that an IHC score of 0 is associated with an expression level below 20,000 receptors per cell, while a score of 1+ corresponds to the expression of 100,000 receptors per cell 52. In preclinical studies, tumor xenografts expressing 40,000 receptors per cell could be clearly visualized when the injected protein dose was low 53, 54. The current study also demonstrated that ^99m^Tc-ZHER2:41071 accumulated in tumors with IHC scores of 2+ or lower. The uptake was characterized by an SUV_max_ equal to 5.0 ± 2.2 in the case of an injected protein dose of 500 µg and 3.6 ± 1.4 in the case of 1000 µg. There was only one patient included in the trial (cohort 500 µg) with an IHC score of 0, and the tumor uptake (SUV = 3.06) was the lowest in this cohort (Figure 8). The small number of patients with very low expression levels in this study does not permit a stringent analysis concerning discrimination between tumors with expression levels of 0 and 1+. A follow-up study is warranted that extends the mass protein dose defined with ^99m^Tc-ZHER2:41071 for discrimination of HER2 low (IHC 2+ or 1+/ISH negative) and HER2 high (IHC 3+ or 2+/ISH positive) to define the optimal dose for fine selection of patients with HER 0 *vs.* HER2 1+ in extended cohorts of patients with HER2-low or negative expression. The data from this work are a good starting point for such a study.

## Conclusions

Injections of ^99m^Tc-ZHER2:41071 appear safe and well tolerated. Dosimetry of ^99m^Tc-ZHER2:41071 permits multiple examinations. Injection of ^99m^Tc-ZHER2:41071 (injected mass 1000 µg) enables discrimination between clinically HER2-positive and HER2-negative (scored by using a standard IHC biopsy test) breast cancer after 2 h. Further clinical development of ^99m^Tc-ZHER2:41071 is warranted.

## Figures and Tables

**Table 1:** Patient Characteristics before Injection with ^99m^Tc-ZHER2:41071

Patient (N)	Age (y)	HER2 status in primary tumor before imaging (ICH/FISH)	Primary tumor status (ER/PgR)	Clinical stage before imaging	Injected activity (MBq)	Primary tumor size (mm)
**500 µg dose of ^99m^Tc-ZHER2:41071**						
1.	44	0	ER+/PR+	IIА (T2N0M0)	377	16*11*25
2.	62	3+	ER-/PR-	IIB (T2N1M0)	325	20*14*22
3.	51	3+	ER+/PR+	IIIC (T4N3M0)	270	39*15*24
4.	59	3+	ER+/PR-	IIА (T2N0M0)	597	21*10*18
5.	48	1+	ER/PR	IIА (T2N0M0)	414	38*30*40
6	55	3+	ER/PR	IIА (T2N0M0)	476	28*23*29
7.	53	2+ FISH negative	ER+/PR+	IIА (T2N0M0)	499	22*11*16
8.	50	1+	ER+/PR+	IIА (T2N0M0)	439	21*14*27
9.	50	1+	ER+/PR-	IIB (T2N1M0)	621	29*21*29
10.	67	3+	ER-/PR-	IIIА (T4N1M0)	479	12*20*20
**1000 µg dose of ^99m^Tc-ZHER2:41071**						
11.	50	3+	ER-/PR-	IIА (T2N0M0)	468	22*27*19
12.	61	3+	ER-/PR-	IIB (T2N1M0)	350	21*14*19
13.	47	2+ FISH negative	ER +/PR +	IIIC (T2N3M0)	570	29*18*30
14.	46	1+	Right breast: ER+/PR+ Left breast: ER+/PR+	Right breast: IIА (T2N0M0) Left breast: IIА (T2N0M0)	438	Right breast: 20*22*24 Left breast: 33*27*38
15.	49	3+	ER-/PR-	IV (T2N2M1)	472	23*19*23,5
16.	62	3+	ER-/PR-	IIIА (T4N0M0)	505	52*41*70
17.	44	1+	ER+/PR+	IIА (T2N0M0)	534	21*17*21
18.	61	1+	ER+/PR+	IIB (T2N1M0)	495	18*13*21
19.	47	3+	ER-/PR-	IIIB (T4N1M0)	434	32*13*24
20.	59	1+	ER+/PR+	IIB (T2N1M0)	427	24*19*19
21.	39	3+	ER-/PR-	IIIC (T2N3M0)	440	34*24*28
**1500 µg dose of ^99m^Tc-ZHER2:41071**						
22.	58	2+ FISH positive	ER+/PR+	IIIB (T4N1M0)	427	23*26*28
23.	36	3+	ER+/PR+	IIА (T2N0M0)	470	15*18*21
24.	55	3+	ER-/PR-	IIА (T2N0M0)	513	21*17*22
25.	48	3+	ER+/PR+	IIB (T2N1M0)	446	25*12*26
26.	51	1+	ER+/PR+	IIА (T2N0M0)	366	23*21*29
27.	57	1+	ER-/PR-	IIА (T2N0M0)	430	33*24*33
28.	66	1+	ER-/PR-	IIА (T2N0M0)	400	14*16*21
29.	39	1+	ER-/PR-	IIА (T2N0M0)	448	21*16*16
30.	51	3+	ER+/PR+	IIА (T2N0M0)	417	37*29*44
31.	60	1+	ER+/PR+	IIА (T2N0M0)	313	24*22*24

**Table 2:** Organs with the Highest Uptake (%ID/organ, decay corrected)

	2 h			4 h			6 h			24 h		
	500	1000	1500	500	1000	1500	500	1000	1500	500	1000	1500
Breast	6.5 ± 2.2	5.6 ± 1.3	6.0 ± 1.2	5.7 ± 1.7	4.9 ± 1.1	4.9 ± 0.7	5.3 ± 1.5	4.1 ± 1.1	4.2 ± 0.7	4.2 ± 2.2 *	3.0 ± 1.0	2.6 ± 0.7
Kidney	17.4 ± 8.1	15.5 ± 3.5	15.6 ± 5.3	15.9 ± 6.0	15.8 ± 3.6	15.2 ± 5.1	16.1 ± 7.1	15.1 ± 4.4	15.4 ± 5.5	18.3 ± 9.5	115.4 ± 4.0	13.9 ± 3.1
Liver	13.9 ± 7.8	11.7 ± 1.9	9.8 ± 2.1	15.4 ± 9.2	11.9 ± 2.7	9.4 ± 2.2	15.4 ± 9.6	11.0 ± 2.9	9.0 ± 2.5	12.7 ± 12.4	9.9 ± 3.9	7.0 ± 2.9
Lungs	5.4 ± 2.4	6.3 ± 1.5	7.0 ± 1.4	6.0 ± 2.1	5.4 ± 1.4	5.9 ± 1.0	5.7 ± 1.7	4.5 ± 1.1	5.1 ± 0.8	4.3 ± 2.8	3.5 ± 1.2	2.9 ± 0.9

*Significant difference (P < 0.05, one-way ANOVA) between patients injected with 500 and 1500 µg ZHER2:41071.

**Table 3:** Absorbed Doses after Injection of Different Masses ^99m^Tc-ZHER2:41071

	500 µg	1000 µg	1500 µg
Adrenals	0.05 ± 0.02^ a^	0.06 ± 0.02	0.07 ± 0.02
Brain	0.0019 ± 0.0003	0.0018 ± 0.0003	0.0020 ± 0.0002
Breasts	0.019 ± 0.004	0.016 ± 0.004	0.016 ± 0.002
Gall bladder Wall	0.029 ± 0.010	0.027 ± 0.010	0.029 ± 0.007
Lower large intestine wall	0.011 ± 0.003	0.012 ± 0.003	0.012 ± 0.002
Small Intestine	0.009 ± 0.002	0.009 ± 0.002	0.009 ± 0.002
Stomach Wall	0.011 ± 0.002^ a^	0.012 ± 0.003	0.014 ± 0.002
Upper large intestine wall	0.012 ± 0.003	0.012 ± 0.003	0.012 ± 0.002
Heart Wall	0.008 ± 0.001	0.008 ± 0.002	0.008 ± 0.001
Kidneys	0.07 ± 0.02	0.07 ± 0.02	0.07 ± 0.02
Liver	0.02 ± 0.01	0.018 ± 0.004	0.015 ± 0.003
Lungs	0.011 ± 0.002	0.010 ± 0.002	0.011 ± 0.001
Muscle	0.0035 ± 0.0005	0.003 ± 0.001	0.00036 ± 0.0005
Ovaries	0.026 ± 0.008^ a^	0.030 ± 0.009^ b^	0.040 ± 0.009
Pancreas	0.024 ± 0.007	0.021 ± 0.005	0.022 ± 0.004
Red Marrow	0.005 ± 0.001	0.004 ± 0.001	0.005 ± 0.001
Osteogenic Cells	0.010 ± 0.001	0.009 ± 0.002	0.010 ± 0.001
Skin	0.0023 ± 0.0003	0.0022 ± 0.0004	0.0024 ± 0.0003
Spleen	0.017 ± 0.007	0.016 ± 0.004	0.016 ± 0.003
Thymus	0.013 ± 0.003^ a^	0.016 ± 0.004	0.019 ± 0.004
Thyroid	0.05 ± 0.01^ a^	0.06 ± 0.01	0.062 ± 0.006
Urinary Bladder Wall	0.01 ± 0.01	0.02 ± 0.02	0.02 ± 0.01
Uterus	0.015 ± 0.004	0.014 ± 0.004	0.015 ± 0.003
Total Body	0.005 ± 0.001	0.005 ± 0.001	0.005 ± 0.001
Effective Dose Equivalent	0.025 ± 0.005	0.026 ± 0.007	0.030 ± 0.005
Effective Dose (mSv/MBq)	0.017 ± 0.003^ a^	0.018 ± 0.005	0.021 ± 0.004

Data are presented as the mean mGy/MBq ± SD. ^a^ Significant (P < 0.05) difference between doses after injection of 500 and 1500 µg. ^b^ Significant (P < 0.05) difference between doses after injection of 1000 and 1500 µg.

**Table 4:** Uptake (SUV) in tumors with different HER2 expression at different time points

	2 h		4 h		6 h	
	HER2-positive	HER2-low/negative	HER2-positive	HER2 low/negative	HER2-positive	HER2 low/negative
500 µg	7.6 ± 2.5	5.0 ± 2.2	8.8 ± 3.2	5.0 ± 2.5	8.9 ± 2.6	4.9 ± 2.4*
1000 µg	16.9 ± 7.6	3.6 ± 1.4**	18.5 ± 8.7	3.8 ± 2.0**	19.8 ± 9.7	3.8 ± 1.3**
1500 µg	8.6 ± 6.1	3.9 ± 2.1	10.2 ± 10.3	3.3 ± 2.1	10.5 ± 10.0	3.4 ± 2.0

*Significant difference (p <0.05, Mann-Whitney U test) between uptake in HER2-positive and HER2-low/negative tumors at this time point. **Highly significant difference (p <0.005, Mann-Whitney U test) between uptake in HER2-positive and HER2-low/negative tumors at this time point.

**Table 5:** Tumor-to-contralateral site ratio for primary tumors with different HER2 expression at different time points

	2 h		4 h		6 h	
	HER2-positive	HER2-low/negative	HER2-positive	HER2 low/negative	HER2-positive	HER2 low/negative
500 µg	21 ± 14	14 ± 10	29 ± 20	15 ± 13	35 ± 15	11 ± 9*
1000 µg	32 ± 18	5 ± 4**	38 ± 18	6 ± 6**	44 ± 20	5 ± 3**
1500 µg	12 ± 7	9 ± 6	16 ± 10	6 ± 4	14 ± 8	8 ± 3

*Significant difference (p <0.05, Mann-Whitney U test) between ratios for HER2-positive and HER2-low/negative tumors at this time point. **Highly significant difference (p <0.005, Mann-Whitney U test) between ratios for HER2-positive and HER2-low/negative tumors at this time point.

**Table 6:** Tumor-to-contralateral site ratio for HER2-positive lymph node metastases at different time points

	2 h	4 h	6 h
500 µg	30 ± 19*	25 ± 11	44 ± 33
1000 µg	9 ± 3*	15 ± 7	16 ± 8
1500 µg	11 ± 12	11 ± 10	23 ± 30

*Significant difference (P < 0.05, one-way ANOVA with Tukey's correction) between lymph node metastasis-to-contralateral site ratios after injection of 500 and 1000 µg.

## Abbreviations

HER2: human epidermal growth factor receptor type 2
IHC: immunohistochemistry
FISH: fluorescent *in situ* hybridization
PET: positron emission tomography
SPECT: single-photon emission computed tomography
ROC: receiver operating characteristic
CT: computed tomography
PSMA: prostate-specific membrane antigen
FAPI: fibroblast activation protein inhibitor
